# Clinical Scenarios for Discordant Anti-Xa

**DOI:** 10.1155/2016/4054806

**Published:** 2016-05-12

**Authors:** Jesus Vera-Aguilera, Hindi Yousef, Diego Beltran-Melgarejo, Teng Hugh Teng, Ramos Jan, Mary Mok, Carlos Vera-Aguilera, Eduardo Moreno-Aguilera

**Affiliations:** ^1^Internal Medicine, Texas Tech University Health Sciences Center at the Permian Basin, 800 W. 4th Street, Odessa, TX 79763, USA; ^2^Texas Tech University Health Sciences Center School of Pharmacy at Dallas, 5920 Forrest Park Lane, Suite 400, Dallas, TX 75235, USA; ^3^University of the Incarnate Word Feik School of Pharmacy, 4301 Broadway, San Antonio, TX 78209, USA; ^4^Departamento de Biología Celular y Tisular, Facultad de Medicina, UNAM, 04510 México, DF, Mexico; ^5^Servicio de Gastrocirugía, Hospital de Especialidades, Centro Médico Nacional Siglo XXI, Instituto Mexicano del Seguro Social, 06720 México, DF, Mexico

## Abstract

Anti-Xa test* measures *the activity of* heparin *against the activity of activated coagulation factor X; significant variability of anti-Xa levels in common clinical scenarios has been observed.* Objective*. To review the most common clinical settings in which anti-Xa results can be bias.* Evidence Review*. Guidelines and current literature search: we used PubMed, Medline, Embase, and MEDION, from 2000 to October 2013.* Results*. Anti-Xa test is widely used; however the assay underestimates heparin concentration in the presence of significant AT deficiency, pregnancy, end stage renal disease, and postthrombolysis and in patients with hyperbilirubinemia; limited published data evaluating the safety and effectiveness of anti-Xa assays for managing UH therapy is available.* Conclusions and Relevance*. To our knowledge this is the first paper that summarizes the most common causes in which this assay can be affected, several “day to day” clinical scenarios can modify the outcomes, and we concur that these rarely recognized scenarios can be affected by negative outcomes in the daily practice.

## 1. Introduction

The anti-Xa assay determines the anticoagulant activity of unfractionated heparin (UFH) by measuring the ability of heparin-bound antithrombin (AT) to inhibit a single enzyme, FXa [1]. In past years, the chromogenic anti-Xa assay has become automated, cost-effective, and more accessible to clinicians. Thus, in many institutions UFH is monitored directly against the Anti-Xa, rather than indirectly via the APTT [2]; different clinical situations can modify the test results including jaundice, hyperlipidemia, and/or hemolyzed samples, resulting in decreased reported anti-Xa levels. Understanding the limitations of the activate partial thromboplastin time (aPTT) and anti-Xa tests used for heparin/low molecular weight heparin (LMWH) monitoring can facilitate anticoagulation management [3]. In this review we review the assays and the most common causes in the clinical setting in which results can be misleading.

*Prothrombin Time Test and International Normalized Ratio*. The prothrombin time (PT) test is the most commonly used method for monitoring oral anticoagulant therapy. The International Normalized Ratio (INR) was adopted in 1982 by converting the PT ratio measured with the local thromboplastin into INR using the International Sensitivity Index (ISI) as the measure of the responsiveness of a PT thromboplastin to the coagulation defect induced by warfarin [4].

Two common laboratory tests, aPTT and anti-Xa, used for anticoagulation monitoring of heparin and LMWHs evaluate different aspects of the coagulation cascade. Anti-Xa tests assess the function of a specific coagulation cofactor, factor Xa, while aPTT tests assess the function of the intrinsic (contact activation) and common coagulation pathways [5]. Understanding the limitations of each analytical test can facilitate interpretation of results and management of anticoagulation.

Abnormal anticoagulation test results may be caused before the start of the assay. These causes are often referred to as “preanalytical errors” [6]. Automated tests with standardized reagent volumes require specific volumes of anticoagulated plasma sample. Typically a 9 : 1 ratio of blood sample to sodium citrate is required. Lower blood-to-citrate ratios dilute coagulation factors, requiring more calcium for citrate effect reversal, and prolong clotting times. Underfilling or overfilling the collection tube will overestimate or underestimate, respectively, the level of anticoagulation [7]. Likewise, patients with polycythemia will have overestimated levels of anticoagulation due to a lower plasma-to-citrate ratio compared with patients with hematocrit values within a normal range [8]. Other common preanalytical errors include contamination of blood samples with exogenous anticoagulants (e.g., heparin-containing catheter and heparin- or EDTA-containing tubes). Quick processing of blood samples (within 3 hours of sample collection) is also important as factors degrade (especially factor VIIIa) and platelets release platelet factor 4 [6]. Platelet factor 4 is thought to neutralize heparin-like molecules in plasma and on endothelial cells [9].

**Figure 1:**
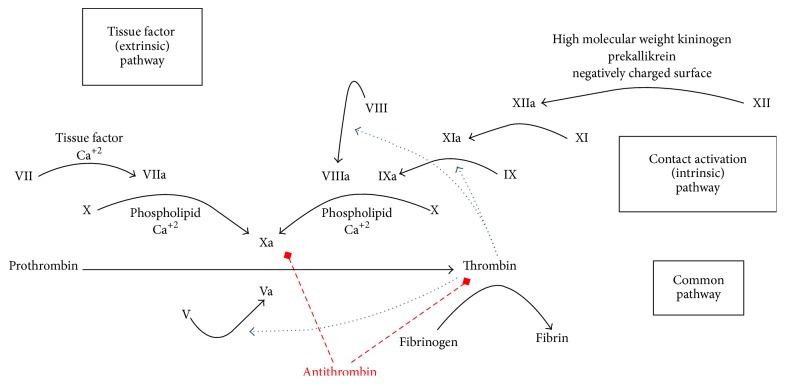
Coagulation cascade.

*Factor Xa*. Factor Xa is the activated coagulation factor that forms part of the prothrombinase complex, along with factor Va, in the common pathway in coagulation cascade (Figure 1). The prothrombinase complex increases the conversion rate of prothrombin to thrombin. Subsequently, thrombin catalyzes the conversion of fibrinogen to fibrin monomers which then polymerize for thrombus formation. Vascular damage results in the release of tissue factor which catalyzes the activation of factor VIIa, also known as extrinsic tenase, and initiates coagulation. Factor VIIa of the extrinsic (or tissue factor) pathway activates Factor Xa. The intrinsic (or contact) pathway propagates coagulation. Factor IXa binds to factor VIIIa on surfaces of activated platelets to form the intrinsic tenase complex [5].

**Figure 2:**
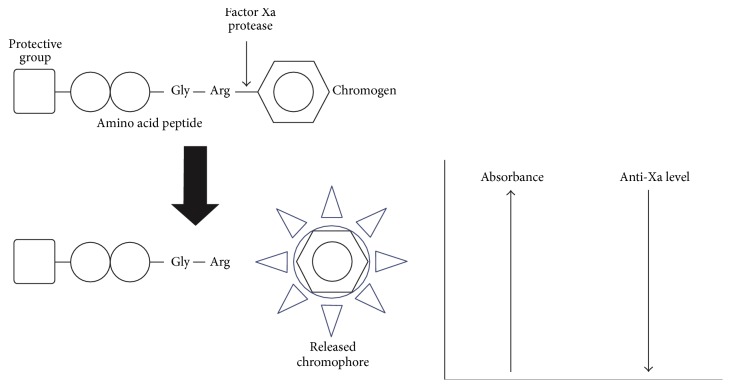
Chromogenic anti-Xa method. Factor Xa cleaves the synthetic chromogenic substrate to release a chromophore, quantified by spectrophotometry absorbance. Absorbance is proportional to factor Xa activity and inversely proportional to anti-Xa level.

*Anti-Xa Test*. Anti-Xa tests, clot-based and chromogenic, were designed to evaluate the anticoagulation effect of heparin based upon inhibition of a single protease, factor Xa [10, 11]. Although the clotting method was developed first, it was considered labor-intensive due to the required sets of serial dilutions to ensure accuracy; the chromogenic method developed by Teien and coworkers in 1976 streamlined the protocol by using a synthetic chromogenic substrate as a marker for factor Xa activity [11]. Teien and Lie later introduced a modified protocol that added purified antithrombin to the plasma sample and improved precision by reducing the effect of patients' endogenous antithrombin concentration variability [12]. In chromogenic assays, factor Xa in the plasma sample cleaves added chromogen substrate to release a colored molecule. A spectrophotometer detects the amount of absorbance from released chromophores, which is proportional to the sample's factor Xa activity (Figure 2). Anticoagulant concentrations and corresponding anti-Xa levels, inversely proportional to spectrophotometer absorbance, are then calculated by comparison to standardized heparin/LMWH curves [11, 12]. The original method developed in 1976 is often referred to as “one-stage” while the later method that adds antithrombin to plasma samples is called “two-stage” [13]. Chromogenic methods are used routinely, and vendors have automated methods and standardized reagent kits [13, 14]. A limitation of chromogenic anti-Xa tests, known since introduction in 1976, is opacity of plasma samples [11]. Icteric, lipemic, and/or hemolyzed samples can interfere with chromogenic methods, resulting in decreased reported anti-Xa levels. Vendors have varying maximum acceptable levels of bilirubin (10–20 mg/dL), triglycerides (600–1,250 mg/dL), and hemoglobin (2 mg/mL) in plasma samples [13, 14]. Recently, researchers found decreased anti-Xa levels in patients on heparin/LMWH therapy with comorbidities of liver diseases and cirrhosis (likely due to reduced synthesis of antithrombin) [15, 16]. However, two-stage chromogenic assays, which account for endogenous antithrombin variability, are not as prevalent as one-stage because the manual addition of exogenous antithrombin is required [13, 14].

## 2. Clinical Scenarios for Discordant Anti-Xa Values

### 2.1. End Stage Renal Disease

LMWH is the preferred treatment for thrombosis in hospital settings; however uncertainty still surrounds use of LMWH in patients with severe renal insufficiency because it is excreted by the kidneys and, unlike UFH, its anticoagulant effect cannot be completely reversed [17, 18]. Observational data of increased bleeding complications have been reported when LMWH is used in patients with chronic renal insufficiency [19, 20]. However, it has been demonstrated in dialysis-dependent patients with a creatinine clearance of 30 mL/min or less treated with standard therapeutic doses of enoxaparin, elevated levels of anti-Xa, and increased risk for major bleeding. Empirical dose adjustment of enoxaparin may reduce the risk for bleeding and merits additional evaluation [18].

Despite this limitation, anti-Xa levels are the only available method to monitor LMWH activity and their use in clinical practice is based on consensus recommendations [18]. Peak anti-Xa levels occur 4 hours after a therapeutic dose of subcutaneous LMWH is administered. Peak levels above the upper limit of the recommended therapeutic range (0.6 to 1.0 IU/mL) may be associated with an increased risk for bleeding in these patients [21].

### 2.2. Morbid Obesity

Morbidly obese patients are often poorly represented or excluded from pharmacokinetic studies and clinical trials. Morbidly obese patients (body mass index [BMI] > 40 kg/m^2^) require unique considerations when selecting medication therapy and dosing strategies. Low molecular weight heparins (LMWHs) are weight-based medications and several considerations must be taken into account when prescribing drug therapy for patients with morbid obesity [22]. Safety and efficacy issues may arise when these are dosed incorrectly [23, 24]. Enoxaparin appears to have a dose response as it relates to anti-Xa activity based on weight up to about 144 kg, with minimal data for dosing enoxaparin in patients above this weight limit [23–27].

Current recommendations for the use of enoxaparin for therapeutic anticoagulation in obese patients include dosing based on actual body weight for therapeutic anticoagulation and adjusting doses based on monitoring of anti-Xa levels [23, 24]. However, data supporting these recommendations are limited, particularly with respect to assessing the doses that patients with morbid obesity require obtaining goal anti-Xa values [22].

### 2.3. Pregnancy

The risk of venous thromboembolism increases in pregnancy, and this risk is mediated by a history of thrombophilia and/or prior thrombotic event. Thrombophilias are also associated with adverse fetal outcomes including intrauterine growth restriction (IUGR), intrauterine fetal demise, severe early-onset preeclampsia, and placental abruption [28].

Heparin prophylaxis is recommended for pregnant patients with a history of thromboembolism, and many experts recommend prophylaxis for pregnant patients with a known thrombophilia and history of adverse pregnancy outcomes associated with these hypercoagulable states [28, 29]. Currently, unfractionated heparin and low molecular weight heparin are considered acceptable for venous thromboembolism prophylaxis during pregnancy, because both are effective in reducing the risk of venous thromboembolism and neither crosses the placenta [30].

Two recently published studies demonstrated that plasma anti-factor Xa levels during pregnancy were lower than expected, indicating that many pregnant patients are receiving subprophylactic dosing [31, 32]. Fox et al. in 2008 analyzed 10 321 anti-factor Xa levels obtained in pregnant woman, from which only 59% were in the prophylactic range. Twenty-six percent of the values were subprophylactic, and 15% were supraprophylactic. When stratified by gestational age, the proportions of prophylactic, subprophylactic, and supraprophylactic values were similar in each trimester [28]. This is an alarming finding, because patients who would normally be given a therapeutic dose have a higher risk of venous thromboembolism during pregnancy, and subtherapeutic dosing could have significant consequences [28].

### 2.4. Antithrombin Deficiency

Antithrombin is a member of the family of serine protease inhibitors (serpins) and is an important natural inhibitor of thrombin, factor Xa, factor IXa, and several other proteases [33]. In normal conditions AT binds to its target proteases acting as a substrate and blocking the active site of the protease. In the case of thrombin, AT binds covalently to it in a 1 : 1 fashion inhibiting its action [34].

Heparin binds to AT causing a conformational change that exposes its active site, increasing the rate of the reaction between AT and thrombin up to 1000-fold, thereby increasing its anticoagulant effect [35]. As AT is required for the anticoagulant action of heparin, it is one of the factors also required for the anti-Xa heparin assay and should be considered when analyzing possible flaws of the test. In conditions where there is a deficiency of AT, heparin levels may actually be more elevated than those the test suggests.

Besides the inherited AT deficiency where the levels can drop down until reaching 50% [36], it is now recognized that certain clinical conditions are associated with an acquired deficiency of AT. Studies in acutely ill patients in the ICU have demonstrated an AT deficit of up to 45% [37] and even less than 30% [38]. In sepsis patients the plasma levels of antithrombin are markedly decreased due to the interaction of several factors including a negative acute phase response and impaired liver function, degradation by neutrophil elastase, and AT consumption [39]. In liver cirrhosis the hepatic synthesis of coagulation factors including antithrombin is also impaired, affecting anti-Xa testing [15]. In the same way cardiopulmonary bypass, nephrotic syndrome, pregnancy, and treatment of acute lymphoblastic leukaemia with asparaginase are settings where a deficiency of AT has been documented [36].

The supplementation of antithrombin to the anti-Xa assay may avoid potential interferences, and it has been demonstrated that assays supplemented in this way have improved heparin recovery, specially when the levels of AT have dropped below 40% [38]; however the clinical utility of these tests is still to be proved.

### 2.5. Jaundice

Bilirubin is an intrinsic chromogenic molecule. Elevated levels of bilirubin can be caused by many diseases discussed elsewhere. The mechanism of the assay depends on spectrophotometry which detects chromogenic agents including bilirubin. Elevated levels of bilirubin can result in underestimation of LMWH and UFH activity. In contrast to hypertriglyceridemia, which can affect the assay through the same mechanism, hyperbilirubinemia cannot be solved by ultracentrifuge. Nor can the sample be redrawn as the disease process is ongoing.

## 3. Discussion

Despite many limitations of Anti-Xa levels, it is the only available method to monitor LMWH activity and their use in clinical practice is based on consensus recommendations [18].

It is important for clinicians and laboratorians to recognize that laboratory data, although potentially extremely useful in diagnostic decision making, should be used as an aid and adjunct to the constellation of findings (e.g., history and physical exam) relevant to the patient. Laboratory data is never a substitute for a good physical exam and patient history (clinicians should treat the patient, not the laboratory results).

In pregnancy, no clear recommendations exist for use of prophylactic low molecular weight heparin in pregnancy [4]. Physiologic changes in normal pregnancy, including weight gain, increased renal clearance, and volume of distribution, may decrease the availability of low molecular weight heparin or produce a less predictable response in pregnant women compared with nonpregnant women. Regarding this, the American College of Obstetricians and Gynecologists states that “because of the lack of data regarding adequate dosing during pregnancy, anti-factor Xa levels may be monitored [29].”

In several observational studies in end stage renal disease, patients treated with standard therapeutic doses of enoxaparin presented an increased risk for major bleeding and elevated levels of anti-Xa. Anti-Xa levels are the only available method to monitor LMWH activity and their use in clinical practice is based on consensus recommendations [18]. In these cases, empirical dose adjustment of enoxaparin is recommended to reduce the risk for bleeding [18].

Safety and efficacy issues arise when weight-based medications are dosed incorrectly or in cases where there minimal data is available as in patients with weight up to 144 kg. Current recommendations for the use of enoxaparin for therapeutic anticoagulation in obese patients include dosing based on actual body weight for therapeutic anticoagulation and adjusting doses based on monitoring of anti-Xa levels [23, 24]. Data supporting these recommendations are limited, particularly with respect to assessing the doses that patients with morbid obesity require to obtain goal anti-Xa values.

## 4. Conclusion

The are several factors in favor of the use of the anti-Xa assay; for example, it is available on many automated coagulation analyzers; the probe is not affected by underfilled collection tubes; it is not susceptible to interference from elevated concentrations of factor VIII or fibrinogen that result from acute phase reactions [40].

However there are disadvantages in the use of the anti-Xa assay: The anti-Xa sample processing (1 hour) is required to avoid heparin neutralization from platelet factor 4; it is more expensive than the PTT and the assay underestimates heparin concentration in the presence of significant AT deficiency, although the clinical significance of this finding is controversial; anti-Xa level can also be affected by pregnancy, end stage renal disease, postthrombolysis, and bilirubin levels; even more there are limited published data evaluating the safety and effectiveness of anti-Xa assays for unfractionated heparin therapy [38, 40].

To our knowledge, this is the first paper that summarizes most of the common causes in which this test can be affected and most of clinicians should be aware that several factors can modify the outcomes in the daily clinical setting and this could be reflected in detriment of patients' safety.
